# Sustainable Whey-Based Functional Beverages Enriched with Andean Blueberry (*Vaccinium floribundum* Kunth) and Blueberry (*Vaccinium corymbosum* L.): Optimization, Antioxidant Properties, and Gastrointestinal Bioaccessibility

**DOI:** 10.3390/foods15111895

**Published:** 2026-05-27

**Authors:** Ociel Muñoz-Fariña, Alba-Paola Maldonado, Olga García Figueroa, Gabriela M. Bulnes-Vides, Luisbel González, María Cristina Ravanal

**Affiliations:** 1 Instituto de Ciencia y Tecnología de los Alimentos, Facultad de Ciencias Agrarias y Alimentarias, Universidad Austral de Chile, Valdivia 5090000, Chile; ocielmunoz@uach.cl (O.M.-F.); paola.maldonadoch@gmail.com (A.-P.M.); olga.garcia@uach.cl (O.G.F.); 2 Departamento Químico Biológico, Universidad Nacional Autónoma de Honduras, Comayagua 12101, Honduras; gabriela.bulnes@unah.edu.hn; 3 Departamento de Química Biológica, Universidad de Sevilla, 41012 Sevilla, Spain; 4 Instituto de Ciencias Aplicadas, Facultad de Ingeniería, Universidad Autónoma de Chile, Santiago 8581151, Chile; 5 Facultad de Agronomía y Sistemas Naturales, Pontificia Universidad Católica de Chile, Santiago 7820436, Chile; 6 Instituto para el Desarrollo Sustentable, Pontificia Universidad Católica de Chile, Santiago 7820436, Chile

**Keywords:** functional beverages, whey valorization, fermented whey, *Vaccinium floribundum*, antioxidant capacity, in vitro digestion, bioaccessibility

## Abstract

Dairy whey is an underutilized by-product with potential as a sustainable carrier for bioactive compounds. This study developed and optimized fermented whey-based beverages enriched with Andean blueberry (*Vaccinium floribundum* Kunth) and blueberry (*Vaccinium corymbosum* L.) extracts and evaluated their antioxidant properties and gastrointestinal bioaccessibility. Beverages were formulated with fermented whey and berry extracts and optimized using a 3ᵏ response surface design considering extract concentration and storage time. The optimal formulations contained 50% berry extract. The Andean blueberry beverage showed the highest functional performance, with 2268.97 ± 4.41 µmol Trolox equivalents (TE)/100 mL by oxygen radical absorbance capacity (ORAC), 1442.46 ± 12.95 µmol TE/100 mL by 2,2-diphenyl-1-picrylhydrazyl radical scavenging assay (DPPH), 242.60 ± 6.25 mg GAE/100 mL of total polyphenols, 137.94 ± 2.76 mg QE/100 mL of flavonoids, and 21.50 ± 0.51 mg C3GE/100 mL of anthocyanins. During in vitro digestion, polyphenols and flavonoids showed high bioaccessibility, reaching values above 80% in gastric or intestinal stages, while ORAC antioxidant capacity increased up to 153% in the jejunal phase. Anthocyanins remained more stable under gastric conditions but decreased during intestinal digestion. These findings support fermented whey as a value-added matrix for developing bioactive-rich functional beverages with improved digestive functionality.

## 1. Introduction

Functional foods and beverages have gained increasing attention as dietary strategies capable of providing health-related benefits beyond basic nutrition. These products are designed to deliver nutrients or bioactive compounds that may contribute to physiological functions associated with oxidative stress, inflammation, metabolic regulation, and chronic disease prevention [1,2]. Among functional ingredients, plant-derived bioactive compounds, particularly phenolics, flavonoids, anthocyanins, carotenoids, and vitamins, are of special interest because of their antioxidant capacity and potential role in modulating cellular and biochemical processes related to human health [3,4].

Berries are widely recognized as rich sources of phenolic compounds, especially anthocyanins, which are hydrophilic pigments responsible for the characteristic red, purple, and blue coloration of these fruits. Anthocyanins have been associated with free radical scavenging activity and protection against oxidative damage, making berries suitable ingredients for the development of functional food matrices [5]. Blueberry (*Vaccinium corymbosum* L.) is one of the most studied berries because of its nutritional value and high content of bioactive compounds, including ascorbic acid, flavonols, hydroxycinnamic acids, hydroxybenzoic acids, stilbenes, and anthocyanins [6,7]. These compounds have been linked to antioxidant, anti-inflammatory, and metabolic regulatory effects, supporting the use of blueberries in functional food development.

In addition to cultivated blueberries, wild Andean berries represent promising but less explored sources of bioactive compounds. Andean blueberry (*Vaccinium floribundum* Kunth), also known as mortiño, grows naturally in high-altitude Andean ecosystems and is traditionally used in Ecuador and Colombia for the preparation of regional beverages such as “Colada Morada”. This species has attracted scientific interest because of its high phenolic content and distinctive anthocyanin profile, particularly delphinidin and cyanidin derivatives [8,9]. Despite its functional potential, studies addressing the incorporation of *V. floribundum* into technologically viable food matrices and evaluating the stability of its bioactive compounds during digestion remain limited.

Importantly, *V. floribundum* differs from conventional cultivated blueberry not only in its botanical origin but also in its functional and market potential. As an underutilized Andean berry, it represents a regional biodiversity resource with a distinctive phenolic composition, particularly a delphinidin-rich anthocyanin profile and high total phenolic and flavonoid contents. These attributes may provide compositional advantages over widely commercialized blueberry products, while supporting the development of value-added functional foods based on native Andean ingredients. Therefore, its incorporation into fermented whey beverages offers a dual opportunity: valorizing a dairy by-product and promoting a less-explored berry species with nutritional, technological, and market differentiation potential.

A suitable matrix for incorporating berry-derived bioactive compounds is dairy whey, an abundant by-product of cheese manufacture. Although whey is increasingly used by the food industry because of its nutritional and technological value, large volumes are still generated during cheese production, and its management remains relevant from an environmental and sustainability perspective due to its high organic load. Whey contains lactose, soluble proteins, minerals, and bioactive peptides, which make it an attractive ingredient for the formulation of functional beverages [10]. Thus, in the present study, the emphasis is not on demonstrating the already recognized nutritional value of whey, but on its use as a sustainable carrier matrix for berry-derived bioactive compounds and as a strategy for dairy by-product valorization.

Nevertheless, the functional value of a beverage depends not only on its initial concentration of bioactive compounds but also on their stability and bioaccessibility during gastrointestinal digestion. Bioaccessibility refers to the fraction of a compound released from the food matrix during digestion and made available for intestinal absorption. In vitro digestion models are widely used to estimate this process under controlled oral, gastric, and intestinal conditions, providing useful information on the potential nutritional and functional behavior of food products [11,12]. This is particularly relevant for anthocyanins, which are highly sensitive to pH changes, enzymatic activity, and interactions with food matrix components. Dairy proteins may interact with phenolic compounds and influence their stability, release, and antioxidant activity during digestion.

Berry-based products, yogurt-type matrices, and whey-derived beverages have demonstrated considerable potential as functional foods, highlighting the opportunity to integrate bioactive-rich fruits with sustainable dairy systems. In this context, fermented whey represents a versatile matrix for incorporating native berries such as *Vaccinium floribundum* Kunth, an Andean species characterized by a distinctive phenolic and anthocyanin composition. This study developed and optimized fermented whey-based beverages enriched with Andean blueberry and blueberry extracts through response surface methodology, followed by physicochemical, sensory, and functional characterization. In addition, the behavior of phenolic compounds, anthocyanins, and antioxidant capacity was assessed under simulated gastrointestinal conditions. By integrating formulation optimization with the evaluation of bioactive stability and bioaccessibility, this work provides new insights into fermented whey as a carrier matrix and highlights the added value of *V. floribundum* as an underutilized Andean berry with compositional advantages over conventional blueberry for the development of differentiated functional beverages.

## 2. Materials and Methods

### 2.1. Raw Materials

Andean blueberry (*Vaccinium floribundum* Kunth) fruits were obtained from Machachi (Pichincha Province, Ecuador), while cultivated blueberry (*Vaccinium corymbosum* L.) fruits were sourced from Puerto Octay (Los Lagos Region, Chile). The fruits were subjected to convective drying, vacuum-packed, and stored at −20 °C until further use. Pasteurized sweet whey derived from Camembert cheese production was kindly provided by ARTISAN (Valdivia, Los Ríos, Chile). Whey was used as the base matrix for beverage formulation.

### 2.2. Preparation of Berry Extracts

Berry extracts were prepared using an aqueous acidified extraction method. Briefly, dried fruits were ground and mixed with acidified water (0.1% HCl, *v*/*v*) at a solid-to-liquid ratio of 3:5 (*w*/*w*). Acidified extraction was selected because anthocyanins are more stable under acidic conditions, where the flavylium cation form is favored. The use of 0.1% HCl (*v*/*v*) was intentionally applied as a mild acidification condition to improve anthocyanin recovery while avoiding excessive acid-induced hydrolysis or degradation, following previously reported anthocyanin extraction procedures using HCl-acidified solvents [13,14]. This extraction condition was selected based on previous literature and was not independently optimized in the present study. The mixture was macerated and stirred at 2000 rpm for 30 min at room temperature. Subsequently, the extract was centrifuged at 5000 rpm for 5 min, and the supernatant was collected and stored at 4 °C for a maximum of 10 h prior to beverage formulation.

The extracts were characterized in terms of total polyphenol content (TPC); total flavonoid content (TFC); total anthocyanin content (TAC); antioxidant capacity, assessed using the 2,2-diphenyl-1-picrylhydrazyl radical scavenging assay (DPPH) and oxygen radical absorbance capacity (ORAC) assays; and anthocyanin profile, assessed by high-performance liquid chromatography with diode-array detection (HPLC-DAD), following previously reported methodologies for berry-derived phenolics [15].

### 2.3. Preparation of Fermented Whey

Fermented whey was prepared by inoculating pasteurized whey with *Lactobacillus helveticus* (CHOOZIT™ Helveticus LYO 2D, Danisco, Copenhagen, Denmark) at a concentration of 0.5 g/L. The inoculated whey was incubated in sterile Schott^®^ bottles at 42 ± 0.1 °C in a thermoregulated water bath under static conditions for approximately 9 h, until reaching a target pH of 4.5. After fermentation, the whey was cooled and stored at 4 °C until use.

### 2.4. Formulation and Storage Optimization Study

Functional beverages were prepared by mixing fermented whey with each berry extract separately. Response surface methodology (RSM) was applied to optimize formulation and storage conditions, rather than the complete beverage production process. Berry extraction, whey fermentation, and homogenization conditions were kept constant because they had been previously standardized to ensure reproducibility and to isolate the effects of extract concentration and storage time on beverage quality. The independent variables were berry extract concentration (30, 40, and 50%, *v*/*v*) and storage time (1, 4, and 7 days). The response variables included pH, titratable acidity, total polyphenol content, total anthocyanin content, antioxidant capacity determined by ORAC and DPPH assays, and sensory acceptance.

For each response, predictive models were fitted within the experimental domain, and individual optima were initially identified. Subsequently, a multi-response optimization was performed using the desirability function approach, which transforms each response into a dimensionless scale ranging from 0 (completely undesirable response) to 1 (fully desirable response). The individual desirability values were then combined into an overall desirability index using the geometric mean, enabling the simultaneous optimization of physicochemical, functional, and sensory attributes.

The resulting desirability values were interpreted according to predefined qualitative criteria to facilitate comparison among formulations: values close to 1.00 indicate optimal conditions; 0.80–0.99 represent highly desirable responses; 0.63–0.80 indicate acceptable performance; 0.37–0.63 correspond to moderate suitability; 0.20–0.37 reflect low desirability; and values below 0.20 indicate inadequate conditions. This approach allows a balanced selection of the optimal formulation while considering multiple responses simultaneously, as described in classical desirability-based optimization frameworks [16].

### 2.5. Proximate Analysis of Raw Materials and Beverages

The proximate composition of the raw material (sweet whey) and optimized beverages was determined according to standard AOAC methods [17]. Moisture content was determined gravimetrically by drying samples at 105 °C in a forced-air oven (UFE 700, Memmert, Schwabach, Germany) until constant weight, following AOAC Method 925.10. Ash content was measured by incineration in a muffle furnace (Model L3/P, Nabertherm GmbH, Lilienthal, Germany) at 550 °C until complete mineralization, according to AOAC Method 923.03. Protein content was quantified using the Kjeldahl method, applying a nitrogen-to-protein conversion factor of 6.38 for dairy products. Total lipid content was determined using the chloroform–methanol extraction method [18]. All determinations were performed in triplicate, and results were expressed as mean ± standard deviation.

### 2.6. Determination of Total Polyphenol Content (TPC)

Total polyphenol content (TPC) was determined in berry extracts and functional beverages before and after in vitro gastrointestinal digestion using the Folin–Ciocalteu colorimetric method, with minor modifications [19]. Briefly, 40 μL of appropriately diluted sample was mixed with 3.16 mL of distilled water and 200 μL of Folin–Ciocalteu reagent. After homogenization, 600 μL of sodium carbonate solution (Na_2_CO_3_, 20%, *w*/*v*) was added to promote the alkaline reaction medium. The mixture was vortexed and incubated in the dark for 120 min at 20 °C. Absorbance was then measured at 765 nm using a Spectronic^®^ 20 Genesys™ spectrophotometer (Thermo Fisher ScientificInc., Madison, WI, USA). Quantification was performed using a gallic acid calibration curve, and results were expressed as mg gallic acid equivalents per 100 mL of sample (mg GAE/100 mL). All reagents were obtained from Merck (Darmstadt, Germany), and all determinations were carried out in triplicate.

### 2.7. Determination of Total Flavonoid Content (TFC)

Total flavonoid content (TFC) was determined in berry extracts and functional beverages using the aluminum chloride colorimetric assay, following the method described by Shraim et al. (2021) [20], with slight modifications. Briefly, an aliquot of sample was mixed with 1575 μL of deionized water and 75 μL of sodium nitrite solution (NaNO_2_, 5%, *w*/*v*), and the mixture was allowed to react for 6 min at room temperature. Subsequently, 150 μL of aluminum chloride solution (AlCl_3_, 10%, *w*/*v*) was added, followed by incubation for 5 min. Then, 500 μL of sodium hydroxide solution (NaOH, 1 M) was incorporated to develop the final color. Absorbance was measured at 400 nm using a spectrophotometer. Quantification was performed using a quercetin calibration curve, and results were expressed as mg quercetin equivalents per 100 mL of sample (mg QE/100 mL). All determinations were performed in triplicate.

### 2.8. Determination of Total Anthocyanin Content (TAC)

Total anthocyanin content (TAC) was determined in berry extracts and functional beverages before and after in vitro gastrointestinal digestion using the pH differential method [21].

Two buffer systems were prepared: potassium chloride buffer (0.025 M KCl, pH 1.0) and sodium acetate buffer (0.4 M CH_3_COONa, pH 4.5). Each sample was diluted 1:9 (*v*/*v*) with each buffer, mixed thoroughly, and allowed to equilibrate in the dark for 5 min at room temperature. Absorbance was recorded at 510 and 700 nm using a Spectronic^®^ 20 Genesys™ spectrophotometer (Thermo Fisher Scientific Inc., Madison, WI, USA). The absorbance at 700 nm was used to correct for haze or turbidity in the samples.

The absorbance difference (ΔA) was calculated using Equation (1).

(1) ΔA = [(A_510_ − A_700_)_pH1.0_ − (A_510_ − A_700_)_pH4.5_],

Total anthocyanin content was expressed as cyanidin-3-O-glucoside equivalents and calculated using Equation (2).

(2) $$TAC(mg\;C3GE/100\;mL)=\frac{\Delta A\times MW\times DF\times 1000}{\varepsilon \times l}$$

where ΔA is the absorbance difference, MW is the molecular weight of cyanidin-3-O-glucoside (449.4 g/mol), DF is the dilution factor, ε is the molar extinction coefficient of cyanidin-3-O-glucoside (26,900 L/mol·cm), and l is the optical path length of the cuvette (1 cm). All reagents were obtained from Merck (Darmstadt, Germany), and all measurements were performed in triplicate.

### 2.9. ORAC Assay

The oxygen radical absorbance capacity (ORAC) assay was performed to evaluate the peroxyl radical scavenging capacity of berry extracts and functional beverages, following the fluorescein-based method [22]. A fluorescein stock solution (100 μM) was prepared in phosphate buffer (75 mM, pH 7.4) and stored at 4 °C in the dark. A fresh working fluorescein solution (100 nM) was prepared daily by dilution in the same buffer. In a black 96-well microplate, 200 μL of fluorescein working solution was mixed with 40 μL of appropriately diluted sample or Trolox standard solution. Trolox standards were prepared at concentrations of 6, 12, 18, and 24 μM in phosphate buffer. The plate was incubated at 37 °C for 30 min.

The reaction was initiated by adding 50 μL of 2,2′-azobis(2-amidinopropane) dihydrochloride solution (AAPH, 108 mM) prepared in phosphate buffer. Fluorescence was recorded every minute at an excitation wavelength of 485 nm and an emission wavelength of 535 nm using a MAX Gemini microplate spectrofluorometer (Thermo Fisher Scientific Inc., Waltham, MA, USA), maintained at 37 °C. Measurements were continued until fluorescence decreased to less than 5% of the initial value.

The area under the fluorescence decay curve (AUC) was calculated for each sample and standard. ORAC values were obtained from the regression curve generated with Trolox standards and expressed as μmol Trolox equivalents per 100 mL of sample (μmol TE/100 mL). All reagents were purchased from Merck (Darmstadt, Germany), and all determinations were performed in triplicate.

### 2.10. DPPH Assay

Antioxidant capacity was determined using the 2,2-diphenyl-1-picrylhydrazyl radical scavenging assay (DPPH•), following the spectrophotometric method [23]. Briefly, 1 mL of appropriately diluted sample was mixed with 2 mL of DPPH solution in methanol (0.1 mM). The mixture was vortexed for 30 s and incubated in the dark at room temperature for 30 min. Absorbance was then measured at 515 nm using a Spectronic^®^ 20 Genesys™ spectrophotometer (Thermo Fisher Scientific Inc., Madison, WI, USA), with methanol as blank. Quantification was performed using freshly prepared Trolox calibration curves. Results were expressed as μmol Trolox equivalents per 100 mL of sample (μmol TE/100 mL). All determinations were carried out in triplicate.

### 2.11. Sensory Evaluation

Sensory evaluation was performed using a consumer-oriented acceptance test with 26 untrained panelists (aged 18–55 years). Panelists were recruited among regular consumers of dairy- and fruit-based beverages and were included only if they reported no known allergy or intolerance to dairy products or berries. Participants were also instructed to avoid eating, drinking coffee, or using strongly flavored products immediately before the evaluation session. The sensory analysis was conducted under controlled laboratory conditions following the recommendations of ISO 8589 for sensory testing environments [24]. At Universidad Austral de Chile, sensory evaluation studies involving conventional food products are assessed under an institutional risk-based approach to food sensory analysis. According to our institutional regulations, studies involving voluntary adult participants, microbiologically safe food products, and non-invasive sensory procedures are considered minimal-risk activities and therefore do not require formal ethics committee approval or an exemption document. Samples were served refrigerated (8 ± 1 °C) in randomized order using three-digit coded cups to minimize bias. Approximately 30 mL of each beverage was provided to each participant, and water was supplied between samples for palate cleansing.

Panelists evaluated color, aroma, flavor, texture, acidity, sweetness, and overall acceptability using a nine-point hedonic scale, where 1 corresponded to “dislike extremely” and 9 to “like extremely”. These attributes were selected because they represent the main sensory drivers influencing consumer perception and acceptance of fermented whey beverages enriched with berry extracts, particularly visual appearance, fruit-related aroma and flavor, acidity balance, sweetness perception, mouthfeel, and overall liking. The presentation order of samples was randomized according to a balanced incomplete block design to minimize carryover and positional effects.

Individual sensory scores were recorded for each attribute and formulation. Data were expressed as mean ± standard deviation, and differences among formulations were evaluated by analysis of variance (ANOVA). When significant differences were detected, Tukey’s post hoc test was applied at a 95% confidence level (*p* < 0.05). Overall acceptability scores were incorporated as one of the response variables in the desirability-based response surface optimization, allowing simultaneous integration of physicochemical, functional, and consumer acceptance parameters. This approach enabled the selection of formulations with not only high antioxidant potential and phenolic content, but also adequate sensory performance and market-oriented acceptability.

### 2.12. Extraction and HPLC-DAD Analysis of Anthocyanins from Beverages

Anthocyanins were extracted from the functional beverages using an acidified solvent partitioning procedure. Briefly, 4 mL of beverage was mixed with 10 mL of acidified methanol containing 0.01% HCl and 5 mL of chloroform. The mixture was shaken in a Rotatubes^®^ system at 1700 rpm for 30 min. Subsequently, 5 mL of chloroform and 5 mL of acidified water containing 0.01% HCl were added, and the mixture was shaken again under the same conditions. The samples were then centrifuged at 5000 rpm for 5 min to promote phase separation.

The upper hydroalcoholic phase, containing the extracted anthocyanins, was collected, filtered through Whatman^®^ No. 1 filter paper, and further centrifuged at 12,000 rpm for 12 min before chromatographic analysis. The clarified extract was used for anthocyanin profiling by HPLC-DAD.

Chromatographic analysis was performed according to Muñoz-Fariña et al. (2023), with minor modifications in the elution program [15]. An Alliance™ 2695 HPLC system coupled to a Waters 2996 photodiode array detector and controlled by Empower^®^ 3 Build 3471 software (Waters Corporation, Milford, MA, USA) was used. Separation was carried out on a Waters XTerra™ C18 column (250 × 5 mm), maintained at 30 °C. The mobile phase consisted of methanol as solvent A and 3% formic acid in water as solvent B. The gradient was programmed from 15% A and 85% B to 25% A and 75% B over 30 min, at a flow rate of 0.8 mL/min. The injection volume was 20 µL, and anthocyanins were monitored at 520 nm.

Anthocyanins were tentatively identified by comparing their retention times and UV–Vis spectra with those previously reported for *Vaccinium* species under similar chromatographic conditions. Quantification was performed using cyanidin-3-O-glucoside as an external standard, and results were expressed as cyanidin-3-O-glucoside equivalents.

### 2.13. In Vitro Gastrointestinal Digestion and Bioaccessibility

In vitro gastrointestinal digestion was performed to evaluate the stability and bioaccessibility of bioactive compounds in the optimized functional beverages under simulated upper gastrointestinal conditions. The assay was carried out according to the procedures described by Tenore et al. (2013) and Minekus et al. (2014), with minor modifications [11,25]. For each beverage, the digestion assay was performed in triplicate using independent digestion vessels. Each replicate started with an initial 40 mL beverage portion, and the sequential addition of simulated digestive fluids was performed in the same vessel throughout the oral, gastric, and intestinal phases. At each sampling point, a 10 mL aliquot was withdrawn from the total digestion mixture present in the vessel at that stage, not from the initial undigested 40 mL beverage. The digestion model included sequential salivary digestion (SD), gastric digestion initial (GDI), gastric digestion final (GDF), and small intestinal digestion stages, corresponding to duodenal (DDM), jejunal (JJM), and ileal (ILM) phases.

For the salivary digestion stage, 40 mL of beverage was mixed with 12 mL of artificial saliva containing double-distilled water, 5.21 mg/mL NaHCO_3_, 0.88 mg/mL NaCl, 0.48 mg/mL KCl, 0.44 mg/mL CaCl_2_·2H_2_O, 1.04 mg/mL K_2_HPO_4_, 2.16 mg/mL mucin, and 0.1 mg/mL α-amylase. The pH was adjusted to 6.8 using 0.1 N HCl. The mixture was homogenized in a stomacher for 3 min, and a 10 mL aliquot was withdrawn from the oral-phase mixture at the end of this stage.

Gastric digestion was simulated by adding 10 mg of pepsin (14,800 U), previously dissolved in 10 mL of 0.1 N HCl. The pH was adjusted to 2.0 using 6 N HCl, and the mixture was incubated at 37 °C for 2 h under agitation at 250 rpm. A 10 mL aliquot was collected immediately after pH adjustment (GDI), and another 10 mL aliquot was collected at the end of the 2 h gastric incubation period (GDF).

For the intestinal phase, the pH of the remaining gastric digest was adjusted to 6.5 using 0.5 N NaHCO_3_. Subsequently, 10 mL of a pancreatin–bile salt solution, prepared by mixing pancreatin (4.0 mg/mL) and bile salts (50.0 mg/mL) at a 1:1 ratio (*v*/*v*), was added. The mixture was incubated at 37 °C for 2 h under orbital shaking at 250 rpm. A 10 mL aliquot was collected after pH adjustment and addition of the intestinal solution (DDM), followed by 10 mL aliquots after 1 h (JJM) and 2 h (ILM) of intestinal digestion.

At each digestion stage, 10 mL aliquots were collected and immediately cooled in an ice bath for 10 min to rapidly reduce enzymatic activity and minimize further digestion reactions before centrifugation. Samples were then centrifuged at 13,000 rpm for 15 min at 4 °C. The supernatants, corresponding to the bioaccessible fractions, were collected and immediately analyzed for total polyphenol content, total flavonoid content, total anthocyanin content, antioxidant capacity, and anthocyanin profile by HPLC-DAD.

The bioaccessibility index (BI) was calculated as the percentage of each bioactive compound, or antioxidant capacity, remaining in the bioaccessible fraction relative to the corresponding non-digested beverage according to Equation (3).

(3) $$BI(\%)=(\frac{C_{EDS}}{C_{FS}})\times 100$$

where $C_{EDS}$ is the concentration of the bioactive compound or antioxidant capacity measured at the end of each digestion stage, and $C_{FS}$ is the concentration or antioxidant capacity measured in the non-digested beverage.

### 2.14. Statistical Analysis

A 3ᵏ response surface design (RSD) was used to optimize the functional beverage formulations. For the in vitro gastrointestinal digestion assay, data were analyzed using a repeated-measures model to evaluate changes across digestion stages. The remaining experimental data were analyzed by analysis of variance (ANOVA). When significant differences were detected, mean comparisons were performed using Tukey’s post hoc test at a 95% confidence level (*p* < 0.05). All experiments were performed in triplicate, and results were expressed as mean ± standard deviation. Statistical analyses and response surface optimization were conducted using Statgraphics Centurion XVII^®^ softwareVersion 17.2.04 (Statgraphics Technologies, Inc., The Plains, VA, USA).

## 3. Results and Discussion

### 3.1. Physicochemical and Functional Characterization of Andean Blueberry and Blueberry Extracts

The physicochemical and functional characterization of *Vaccinium floribundum* Kunth and *Vaccinium corymbosum* L. extracts is presented in Table 1. Significant differences were observed in pH values (*p* < 0.05), with the Andean blueberry extract showing a lower pH (2.91 ± 0.01) than the blueberry extract (3.09 ± 0.01). This difference may be attributed to variations in organic acid composition, mainly citric, malic, quinic, and ascorbic acids, which are strongly influenced by genotype, maturity stage, geographic origin, and postharvest conditions. Similar pH values have been reported for highbush blueberries, where citric and malic acids are major contributors to acidity [26]. In *V. floribundum*, the acidic profile may also reflect adaptation to high-altitude Andean environments, where environmental stress can modulate primary and secondary metabolism [9]. From a technological perspective, this acidic environment is relevant because it favors the flavylium cation form of anthocyanins, contributing to pigment stability, color intensity, and preservation during processing and early gastric digestion.

Both extracts showed high DPPH radical-scavenging activity, with values of 2564.91 ± 11.82 and 2497.91 ± 66.88 µmol TE/100 mL for Andean blueberry and blueberry extracts, respectively, without significant differences. This suggests that both matrices contain compounds with strong electron- or hydrogen-donating capacity. However, ORAC revealed a clearer functional differentiation: the Andean blueberry extract reached 5242.89 ± 86.36 µmol TE/100 mL, approximately 2.7-fold higher than the blueberry extract (1953.89 ± 48.18 µmol TE/100 mL). This divergence between assays is expected, as DPPH reflects the reduction of a stable radical in a simplified chemical system, while ORAC evaluates peroxyl radical inhibition over time through hydrogen atom transfer. Therefore, the superior ORAC response of *V. floribundum* suggests a richer pool of hydrophilic antioxidants with sustained chain-breaking capacity, probably associated with phenolics, flavonoids, anthocyanins, and other reducing compounds. Similar assay-dependent differences have been reported in berry products, where antioxidant capacity depends not only on total concentration but also on molecular structure, reaction kinetics, and matrix composition [6,27].

The total polyphenol content (TPC) confirmed the higher phenolic density of the Andean blueberry extract, which reached 598.92 ± 4.94 mg GAE/100 mL, compared with 323.20 ± 7.37 mg GAE/100 mL in blueberry extract. This represents an approximately 1.85-fold increase and is consistent with previous reports describing Andean *Vaccinium* species as rich sources of hydroxycinnamic acids, flavonols, and anthocyanins [8,28]. Mechanistically, phenolic compounds contribute to antioxidant capacity through single-electron transfer, hydrogen atom transfer, metal chelation, and stabilization of phenoxyl radicals by resonance. However, the Folin–Ciocalteu assay should be interpreted as an estimate of total reducing capacity rather than a fully specific phenolic measurement, since ascorbic acid, reducing sugars, and other redox-active molecules may also contribute to the response.

A similar trend was observed for total flavonoid content (TFC), with values of 406.77 ± 8.59 mg QE/100 mL for Andean blueberry and 184.16 ± 6.08 mg QE/100 mL for blueberry. This result is relevant because flavonoids, including quercetin, myricetin, and their glycosylated derivatives, are among the phenolic subclasses most closely associated with antioxidant, anti-inflammatory, and metabolic regulatory properties in *Vaccinium* fruits [2,7]. Their activity depends strongly on hydroxylation pattern, conjugation, glycosylation, and the presence of catechol-type structures, which favor electron delocalization and radical stabilization. Thus, the high flavonoid content of *V. floribundum* likely contributed substantially to its higher ORAC response.

Interestingly, total anthocyanin content (TAC) did not follow the same trend as TPC and TFC. The blueberry extract showed a slightly higher TAC value (34.68 ± 0.77 mg C3GE/100 mL) than the Andean blueberry extract (32.71 ± 0.77 mg C3GE/100 mL). This indicates that the superior antioxidant performance of *V. floribundum* was not driven exclusively by anthocyanin concentration, but rather by the broader phenolic matrix and the qualitative anthocyanin profile. HPLC-DAD analysis showed that the Andean blueberry extract was dominated by delphinidin derivatives, mainly delphinidin-3-arabinoside (14.47 ± 0.01 mg C3GE/100 mL) and delphinidin-3-glucoside (10.64 ± 0.01 mg C3GE/100 mL). In contrast, the blueberry extract was characterized mainly by cyanidin-3-glucoside (13.03 ± 0.01 mg C3GE/100 mL), followed by cyanidin-3-arabinoside (5.29 ± 0.01 mg C3GE/100 mL) and delphinidin-3-galactoside (5.10 ± 0.01 mg C3GE/100 mL). This difference is mechanistically important because delphinidin has three hydroxyl groups in the B ring, whereas cyanidin has two; greater hydroxylation generally enhances radical-scavenging capacity but may also increase susceptibility to oxidation and degradation under neutral or mildly alkaline conditions. Similar delphinidin-rich profiles have been reported for mortiño and related Andean *Vaccinium* species [8,29].

The relatively moderate TAC values observed in both extracts may be partly explained by the convective drying applied before extraction. Anthocyanins are thermolabile and can undergo glycosidic bond cleavage, hydration of the flavylium cation, chalcone formation, oxidation, and polymerization during thermal processing. These reactions reduce the measurable monomeric anthocyanin fraction but may generate degradation products or polymeric pigments that still contribute to antioxidant capacity. This helps explain why Andean blueberry maintained high TPC, TFC, and ORAC despite having TAC values comparable to blueberry. The effect of heat on phenolic stability and polyphenol–matrix interactions has been reported in phenolic-enriched dairy systems [30].

Overall, these results demonstrate that both berry extracts are suitable bioactive ingredients for fermented whey-based beverage formulation. However, *V. floribundum* showed a more advantageous functional profile, characterized by lower pH, higher TPC, higher TFC, and markedly higher ORAC antioxidant capacity. Although *V. corymbosum* showed slightly higher total anthocyanin content, the functional superiority of Andean blueberry appears to be driven by its greater phenolic complexity, flavonoid abundance, and delphinidin-rich profile. These findings provide a strong biochemical basis for selecting *V. floribundum* as a promising native ingredient for the development of antioxidant-rich fermented whey beverages and justify further evaluation of its stability, interaction with whey proteins, and gastrointestinal bioaccessibility.

From a novelty perspective, these findings clearly differentiate *V. floribundum* from the widely studied *V. corymbosum*. Although both species belong to the same genus, the Andean blueberry extract showed markedly higher total polyphenol content, total flavonoid content, and ORAC antioxidant capacity, together with a delphinidin-dominant anthocyanin profile. This compositional pattern suggests that *V. floribundum* is not merely an alternative blueberry source, but a functionally distinct Andean berry with added value for the development of premium, antioxidant-rich beverages. Its use may also contribute to the diversification of berry-based functional products by incorporating native biodiversity into sustainable food innovation.

### 3.2. Optimization of Functional Beverages

The formulation and storage optimization of fermented whey-based beverages was performed using a 3ᵏ response surface design, considering berry extract concentration and storage time as the two experimental factors. Therefore, the optimization should be interpreted as a formulation–storage optimization rather than a full process optimization. Fermentation conditions, extraction solvent ratio, and homogenization treatment were not included as independent variables because they were fixed based on previously standardized laboratory protocols. This strategy allowed the study to focus on the impact of berry extract incorporation and refrigerated storage on physicochemical, functional, and sensory responses. This approach was selected because functional beverage development requires the simultaneous optimization of multiple quality attributes, including antioxidant capacity, phenolic retention, acidity, pH, and sensory acceptability. Response surface methodology has been widely used in food and beverage formulation because it enables the identification of optimal processing or formulation conditions while reducing experimental runs and evaluating interactions among variables [31,32]. In this context, the desirability function is particularly useful because it converts different responses into a common dimensionless scale and integrates them into a single optimization criterion, allowing the selection of a formulation with balanced functional, physicochemical, and sensory performance [33].

**Figure 1:**
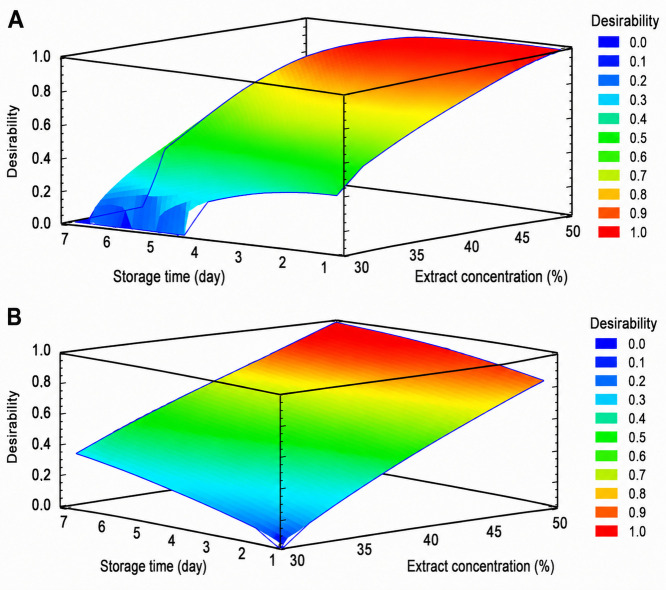
Response surface plots showing the effect of extract concentration and storage time on the overall desirability of fermented whey-based beverages formulated with (**A**) blueberry (*Vaccinium corymbosum* L.) extract and (**B**) Andean blueberry (*Vaccinium floribundum* Kunth) extract.

The response surface plots obtained for both beverages are shown in Figure 1. For the blueberry beverage, the optimal condition was predicted at 50% extract concentration and 1.5 days of storage (Figure 1A). In contrast, the Andean blueberry beverage reached its highest desirability at 50% extract concentration after 7 days of storage (Figure 1B). This contrasting behavior indicates that the two berry extracts interacted differently with the fermented whey matrix during storage. In the blueberry beverage, desirability decreased as storage time increased, suggesting progressive changes in quality attributes such as pigment stability, acidity balance, antioxidant response, or sensory freshness. Similar storage-dependent changes have been reported in anthocyanin-containing dairy systems, where the stability of pigments and phenolic compounds may be affected by pH, oxygen, light exposure, protein interactions, and refrigerated storage time [34]. However, the desirability of the blueberry beverage remained close to 80% at day 7, indicating that the formulation retained acceptable technological and functional performance within the evaluated storage period.

For the Andean blueberry beverage, desirability increased with both extract concentration and storage time, suggesting a more favorable evolution of the system during refrigerated storage. This behavior may be associated with progressive matrix stabilization and improved phenolic solubility during refrigerated storage. Considering the known affinity between dairy proteins and phenolic compounds, matrix interactions could also have contributed to the observed response, although this mechanism was inferred from the behavior of the system and supporting literature rather than directly measured in this study. Whey proteins can interact with anthocyanins and other polyphenols through hydrogen bonding, hydrophobic interactions, electrostatic forces, and, under certain processing conditions, covalent mechanisms. These interactions may modify pigment stability, color expression, antioxidant activity, and the apparent availability of phenolic compounds in dairy matrices [35,36,37]. Therefore, the increase in desirability during storage should not be interpreted simply as an increase in native bioactive concentration, but rather as an improvement in the overall balance among functional activity, physicochemical stability, and sensory perception.

In both beverages, the highest overall desirability was obtained at the highest extract concentration evaluated (50% *v*/*v*). This indicates that, within the experimental range, increasing the proportion of berry extract improved the global formulation response. Similar trends have been observed in fruit-enriched whey or fermented dairy beverages, where the incorporation of fruit-derived bioactive compounds improved antioxidant potential, phenolic content, color attributes, and consumer-oriented quality parameters, although the final response depended strongly on matrix composition and fruit type [38,39]. Nevertheless, high extract incorporation may also increase acidity, astringency, pigment intensity, and phenolic–protein complexation, which can negatively affect sensory quality if not properly balanced. The selection of 50% extract as the optimal formulation therefore suggests that the increase in functional properties did not compromise physicochemical or sensory acceptability within the studied domain.

The opposite storage trends observed for blueberry and Andean blueberry beverages are relevant from a product development perspective. The blueberry formulation appeared more suitable for early consumption, whereas the Andean blueberry beverage showed improved global performance after storage. This difference may reflect the distinct phenolic composition of each extract, particularly the relative abundance and reactivity of anthocyanin subclasses, non-anthocyanin flavonoids, and phenolic acids. In dairy-based systems, phenolic compounds may be stabilized or partially masked by protein interactions, and their antioxidant response can change over time depending on the balance between degradation, release, complexation, and copigmentation phenomena [40,41]. Thus, the storage behavior observed in this study supports the need to optimize each berry–whey system independently rather than assuming that different *Vaccinium* extracts will behave similarly in a fermented dairy matrix.

Overall, the desirability-based optimization demonstrated that fermented whey is a suitable carrier for high levels of berry extracts and that 50% extract concentration provided the best compromise between functionality, physicochemical quality, and sensory performance. The Andean blueberry beverage was particularly promising because its desirability increased during storage, suggesting that this native berry extract may form a more stable or functionally favorable system within fermented whey. These results support the selection of the optimized formulations for subsequent proximate characterization and gastrointestinal bioaccessibility assays.

It should be noted that the response surface design was focused on identifying the most suitable formulation within the selected experimental domain, using extract concentration and storage time as the main variables. Therefore, fermentation, extraction, and homogenization conditions were kept constant to reduce experimental variability and allow a clearer interpretation of the formulation effect. Although an additional independent confirmation run of the predicted optimal point was not performed, the selected formulations were subsequently subjected to extensive compositional, functional, and gastrointestinal bioaccessibility characterization. These complementary analyses provided experimental support for the suitability of the selected formulations and reinforced their relevance as functional whey-based beverages. Future studies may expand this approach by incorporating additional processing variables into the optimization model.

### 3.3. Proximate Characterization of the Beverages

The proximate composition and physicochemical properties of pasteurized whey, fermented whey, and the optimized fermented whey-based beverages are presented in Table 2. Fermentation markedly modified the whey matrix, reducing pH from 6.68 ± 0.01 to 4.52 ± 0.01 and increasing titratable acidity from 1.47 ± 0.06 to 3.41 ± 0.05 g lactic acid/L. This behavior reflects lactose conversion into lactic acid by Lactobacillus helveticus, a key process for improving microbial stability, modifying protein charge, and generating an acidic matrix suitable for fruit-derived phenolics. Similar acidification patterns have been reported in fermented whey beverages, where lactic fermentation improves technological stability and functional potential [42].

After incorporation of berry extracts, pH decreased further to 3.15 ± 0.01 and 3.21 ± 0.00 for the Andean blueberry and blueberry beverages, respectively. This decrease is mainly associated with the organic acid contribution of the fruit extracts. From a technological perspective, this acidic range is favorable for anthocyanin stabilization, since low pH promotes the flavylium cation form, which is more stable and responsible for red–purple coloration. However, acidity must be balanced because it may also influence sensory perception, protein stability, and overall acceptability.

Protein content did not differ significantly between pasteurized and fermented whey, with values of 1.42 ± 0.11 and 1.48 ± 0.27 g/100 mL, respectively. These values are comparable to whey-based fermented systems previously reported in the literature. After beverage formulation, protein decreased to 0.44 ± 0.01 and 0.42 ± 0.02 g/100 mL for Andean blueberry and blueberry beverages, respectively. This reduction was mainly due to the 50:50 dilution of fermented whey with berry extract, rather than protein degradation. Despite this decrease, the beverages retained whey-derived proteins, mainly β-lactoglobulin, α-lactalbumin, serum albumin, immunoglobulins, and glycomacropeptide, which are relevant because they can release bioactive peptides with antioxidant, antimicrobial, immunomodulatory, antihypertensive, and metabolic effects during fermentation or gastrointestinal digestion [1,43].

Total fat also decreased after formulation, from 1.52 ± 0.31 g/100 mL in fermented whey to 0.38 ± 0.02 and 0.43 ± 0.02 g/100 mL in the Andean blueberry and blueberry beverages, respectively. This compositional change supports the development of low-fat functional beverages, while the reduced lipid fraction may also influence mouthfeel and aroma release. Similar low-fat profiles have been reported in fruit-enriched whey products, including whey-based frozen desserts and beverages, where fruit incorporation modifies the balance between nutritional composition, texture, and sensory quality [44].

The addition of berry extracts substantially modified the carbohydrate and mineral profiles. Carbohydrates increased from 3.66 ± 0.01 g/100 mL in fermented whey to 11.06 ± 0.06 and 10.00 ± 0.20 g/100 mL in the Andean blueberry and blueberry beverages, respectively. This increase reflects the contribution of fruit-derived soluble solids, mainly sugars and organic acids, which may improve sweetness, body, and palatability. In parallel, ash content increased from 0.28 ± 0.03 g/100 mL in fermented whey to 0.56 ± 0.02 and 0.45 ± 0.02 g/100 mL in the Andean blueberry and blueberry beverages, respectively, indicating an additional mineral contribution from the fruit extracts. Berries are known to provide minerals such as potassium, calcium, magnesium, phosphorus, iron, copper, and zinc, although their levels depend on genotype, soil composition, geographic origin, and postharvest handling [8,45].

Lactose decreased from 3.18 ± 0.01 g/100 mL in pasteurized whey to 1.86 ± 0.05 g/100 mL after fermentation, confirming partial lactose utilization by *L. helveticus*. In the final beverages, lactose was further reduced to 0.97 ± 0.06 and 0.99 ± 0.01 g/100 mL for Andean blueberry and blueberry formulations, respectively, mainly due to dilution with the fruit extracts. This reduction is nutritionally relevant, as fermented whey beverages with lower lactose content may be better tolerated than non-fermented dairy matrices by some consumers. In addition, lactose may contribute to calcium absorption and to the nutritional value of dairy-based beverages [46].

Fructose and glucose were detected only in the berry-containing beverages, confirming their fruit-derived origin. The Andean blueberry beverage showed higher fructose content (4.48 ± 0.14 g/100 mL), whereas the blueberry beverage showed higher glucose content (3.88 ± 0.02 g/100 mL). These differences are relevant for sensory perception, since fructose has a higher relative sweetness than glucose. Therefore, the sugar profile may have contributed to the higher desirability observed for formulations containing 50% extract during optimization.

Overall, the optimized beverages showed a differentiated composition compared with fermented whey alone: lower pH, reduced protein and fat contents due to dilution, increased carbohydrates and minerals from berry extracts, and lower lactose levels due to fermentation and formulation effects. These changes demonstrate that fermented whey can act as a suitable carrier matrix for berry-derived compounds, combining dairy-derived proteins and residual lactose with fruit-derived sugars, minerals, organic acids, and bioactive compounds. This compositional profile provides the basis for interpreting the functional properties and gastrointestinal behavior of the optimized beverages in subsequent sections.

### 3.4. Characterization of Functional Components in Andean Blueberry and Blueberry Beverages

The functional composition of the optimized fermented whey-based beverages is presented in Table 3. Significant differences were observed between formulations (*p* < 0.05), with the Andean blueberry beverage showing the highest antioxidant capacity, reaching 1442.46 ± 12.95 μmol TE/100 mL by DPPH and 2268.97 ± 4.41 μmol TE/100 mL by ORAC. Although these values were lower than those of the corresponding extracts, mainly due to dilution after incorporation into the fermented whey matrix, both beverages retained substantial antioxidant potential. This indicates that the optimized 50% extract formulation was effective in transferring berry-derived antioxidant compounds into the dairy matrix without a disproportionate loss of functional activity.

The higher antioxidant capacity of the Andean blueberry beverage was consistent with its higher total polyphenol and flavonoid contents. TPC reached 242.60 ± 6.25 mg GAE/100 mL in the Andean blueberry beverage, compared with 178.74 ± 5.54 mg GAE/100 mL in the blueberry beverage. Similarly, TFC was higher in the Andean blueberry beverage (137.94 ± 2.76 mg QE/100 mL). These results suggest that non-anthocyanin phenolics, particularly flavonols and phenolic acids, contributed substantially to the antioxidant response. Polyphenols act through complementary mechanisms, including electron transfer, hydrogen atom transfer, metal chelation, and stabilization of phenoxyl radicals, which may explain the strong association between TPC, TFC, and ORAC response. Similar relationships between phenolic content and antioxidant activity have been reported in berry-derived matrices and fruit-enriched dairy systems [6,8,44].

The antioxidant activity of the beverages should not be attributed exclusively to fruit phenolics. Fermented whey may also contribute through bioactive peptides released during fermentation or later during digestion. Whey proteins, especially β-lactoglobulin and α-lactalbumin, contain peptide sequences with antioxidant potential, which can act by scavenging radicals, chelating transition metals, or donating protons/electrons. Therefore, the final antioxidant response of the beverages likely reflects the combined contribution of berry phenolics and whey-derived components. In this context, protein–polyphenol interactions may help explain changes in the apparent antioxidant response, as reported for similar dairy–phenolic systems. However, in the present study, this interpretation should be considered an inference based on the observed functional behavior and previous literature [1,47].

Total anthocyanin content also differed significantly between beverages. The Andean blueberry beverage showed the highest TAC value, reaching 21.50 ± 0.51 mg C3GE/100 mL. This result indicates that, despite dilution in the whey matrix, a considerable fraction of anthocyanins was retained in the optimized beverage. The TAC values obtained were higher than those previously reported for some fruit-enriched fermented dairy products, such as strawberry-enriched yogurt, where values of 1.86–2.99 mg C3GE/100 g were observed [48]. This comparison supports the effectiveness of the berry–whey formulation as a carrier system for anthocyanin-rich ingredients.

The HPLC-DAD profile revealed clear differences in anthocyanin composition between both beverages. In the Andean blueberry beverage, the predominant anthocyanins were delphinidin-3-arabinoside and delphinidin-3-glucoside, with values of 8.96 ± 0.46 and 7.94 ± 0.08 mg C3GE/100 mL, respectively. In contrast, cyanidin-3-glucoside was the major anthocyanin in the blueberry beverage, reaching 7.95 ± 0.18 mg C3GE/100 mL. These differences are relevant because anthocyanin bioactivity and stability depend not only on total concentration but also on aglycone structure and glycosylation pattern. Delphinidin derivatives, characterized by a higher hydroxylation degree in the B ring, may provide stronger radical-scavenging potential, although they are generally more susceptible to oxidation and pH-induced degradation than less hydroxylated anthocyanins. Cyanidin derivatives, in contrast, may present different stability and color behavior depending on the food matrix and storage conditions [5,29].

The absence or low detectability of cyanidin-3-glucoside and cyanidin-3-arabinoside in the Andean blueberry beverage may be explained mainly by their low initial abundance in the extract and subsequent dilution in the whey matrix. Matrix interactions with whey proteins may also have influenced their apparent recovery, although this should be interpreted as a plausible explanation rather than direct evidence of complex formation. Anthocyanins can form non-covalent complexes with dairy proteins through hydrogen bonding, hydrophobic interactions, and electrostatic interactions. These associations may affect chromatographic recovery, spectral response, color stability, and apparent quantification. Thus, the reduction in detectable monomeric anthocyanins should not necessarily be interpreted as complete degradation, but may also reflect matrix binding or partial transformation into polymeric or copigmented structures.

Overall, the optimized beverages retained a relevant fraction of berry-derived functional compounds after incorporation into fermented whey. The Andean blueberry beverage showed a superior functional profile, characterized by higher DPPH and ORAC antioxidant capacities, higher TPC, higher TFC, higher TAC, and a delphinidin-rich anthocyanin profile. These results suggest that *V. floribundum* is particularly suitable for developing fermented whey-based functional beverages with high antioxidant potential. Moreover, possible matrix interactions between fruit phenolics and whey components may contribute to modulating stability, antioxidant response, and subsequent gastrointestinal bioaccessibility.

### 3.5. Bioaccessibility of the Beverages

The optimized fermented whey-based beverages were subjected to simulated in vitro gastrointestinal digestion to evaluate the release, stability, and bioaccessibility of their functional compounds. This approach is widely used to estimate the fraction of bioactive compounds released from the food matrix and potentially available for intestinal absorption, although it should be interpreted as bioaccessibility rather than true bioavailability [11,49]. In the present study, total polyphenols, flavonoids, anthocyanins, and antioxidant capacity were monitored throughout oral, gastric, and intestinal digestion stages, allowing the evaluation of both compound release and matrix-dependent transformations.

**Figure 2:**
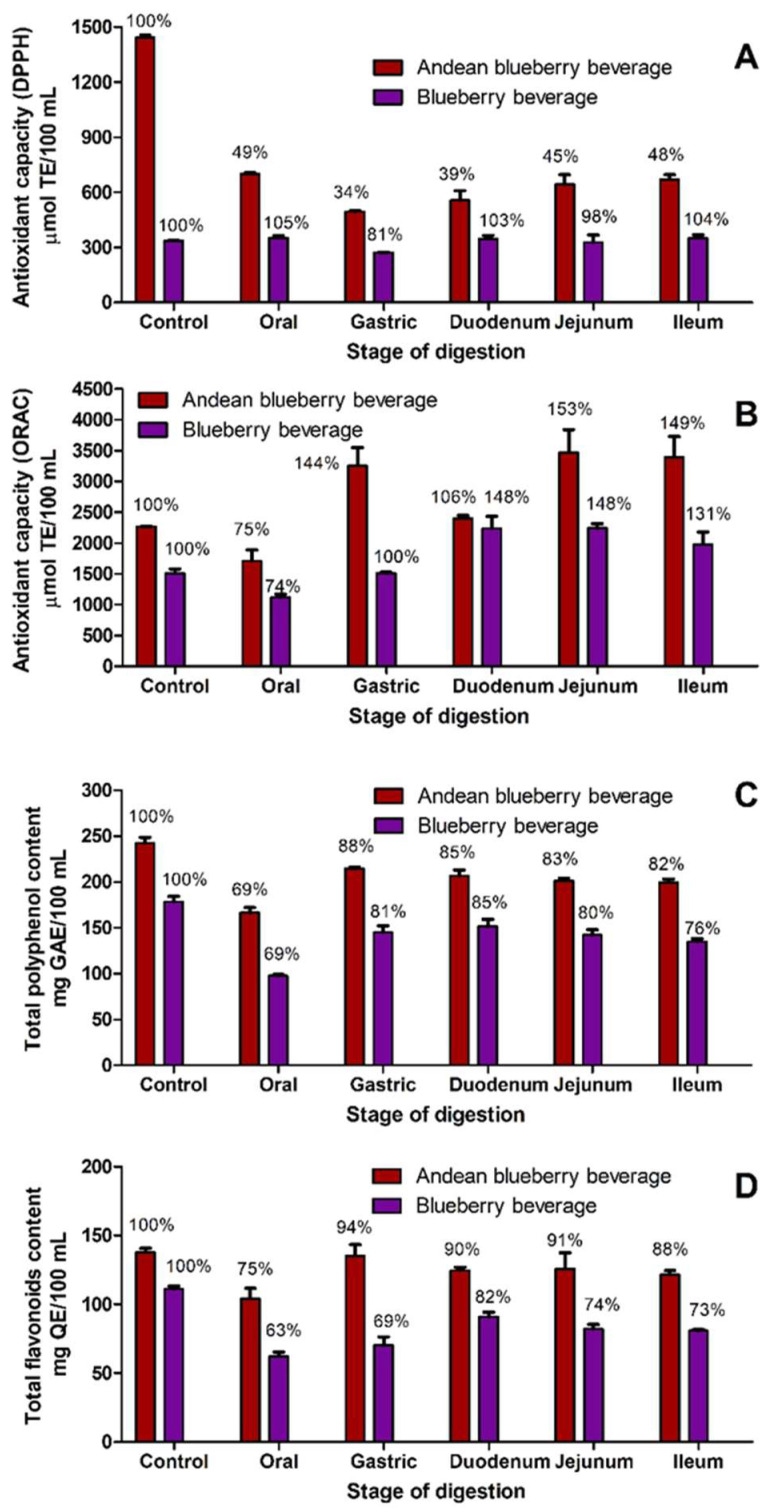
Changes in antioxidant capacity and phenolic compound bioaccessibility during simulated in vitro gastrointestinal digestion of optimized fermented whey-based beverages formulated with Andean blueberry (*Vaccinium floribundum* Kunth) and blueberry (*Vaccinium corymbosum* L.) extracts: (**A**) DPPH antioxidant capacity, (**B**) ORAC antioxidant capacity, (**C**) total polyphenol content, and (**D**) total flavonoid content. Bars represent mean ± standard deviation.

The antioxidant capacity (Figure 2A) measured by DPPH varied significantly across digestion stages (*p* < 0.05), showing a progressive increase during intestinal digestion, especially in the blueberry beverage, which reached 104% bioaccessibility at the final intestinal stage. In contrast, the Andean blueberry beverage showed higher absolute DPPH values but lower final relative bioaccessibility. This difference suggests that the two beverages differed not only in their initial antioxidant load but also in the way their compounds were released, transformed, or detected during digestion. DPPH is based on electron- or hydrogen-donating ability in a relatively simplified chemical system; therefore, changes during digestion may reflect the release of smaller phenolics, degradation products with reducing capacity, or matrix components that become more accessible after enzymatic hydrolysis. Similar increases in antioxidant activity after digestion have been reported in fruit juices and phenolic-enriched fermented dairy matrices [50,51].

The ORAC response (Figure 2B) showed even higher bioaccessibility values, reaching maximum levels in the jejunal phase: 153% for the Andean blueberry beverage and 148% for the blueberry beverage. Final intestinal values remained above 100%, with 149% and 131%, respectively. This result is mechanistically relevant because ORAC measures peroxyl radical scavenging through hydrogen atom transfer over time and can capture the contribution of both phenolic and non-phenolic antioxidants. In fermented whey systems, intestinal digestion can release antioxidant peptides from β-lactoglobulin, α-lactalbumin, and other whey proteins, which may contribute to radical scavenging, metal chelation, and reducing activity. Therefore, the increase in ORAC after digestion may reflect the release of matrix-bound phenolic compounds, the formation of lower-molecular-weight antioxidant metabolites during digestion, changes in compound extractability, and the contribution of digestion-derived whey components [1]. Similar increases above 100% bioaccessibility have been reported in phenolic-rich food systems after simulated gastrointestinal digestion and are commonly associated with enhanced release or transformation of antioxidant compounds under digestive conditions. The possible contribution of antioxidant peptides derived from whey proteins cannot be excluded, although peptide formation was not directly evaluated in the present study.

Total polyphenol content (Figure 2C) also changed significantly during digestion. The Andean blueberry beverage maintained higher TPC values than the blueberry beverage throughout all stages, with maximum values in the gastric and duodenal phases: 214.67 ± 1.89 mg/100 mL (88%) and 206.94 ± 6.36 mg/100 mL (85%), respectively. The blueberry beverage reached its maximum TPC in the duodenal phase, with 151.63 ± 7.86 mg/100 mL (85%). These results suggest that gastric acidification and early intestinal conditions promoted the release of phenolics from the whey–berry matrix. In dairy systems, polyphenols may interact with proteins through hydrogen bonding, hydrophobic interactions, electrostatic forces, and, under some conditions, covalent interactions. These interactions can initially protect phenolics from degradation, while subsequent proteolysis may release bound compounds and increase their measurable bioaccessible fraction [35,37]. Thus, the high TPC recovery observed in the gastric and duodenal phases supports a matrix-mediated protection/release mechanism rather than a simple degradation process.

The behavior of total flavonoids (Figure 2D) followed a similar pattern. The Andean blueberry beverage showed the highest TFC values in all digestion stages, with a maximum in the gastric phase of 129.86 ± 1.68 mg/100 mL, corresponding to 94% bioaccessibility. In the blueberry beverage, the highest TFC was observed in the duodenal phase, reaching 91.11 ± 3.08 mg/100 mL (82%). This suggests that flavonoids were efficiently released under acidic or early intestinal conditions, but their behavior depended on the fruit matrix and its interaction with whey proteins. Flavonoids such as quercetin and myricetin derivatives can bind to proteins through non-covalent interactions, which may reduce immediate detectability but improve stability during digestion. As proteolysis progresses, these compounds may be partially released, increasing their apparent bioaccessibility. Similar protective effects of dairy matrices on phenolic compounds during digestion have been reported for yogurt systems enriched with cinnamon or coffee phenolics [51,52].

**Figure 3:**
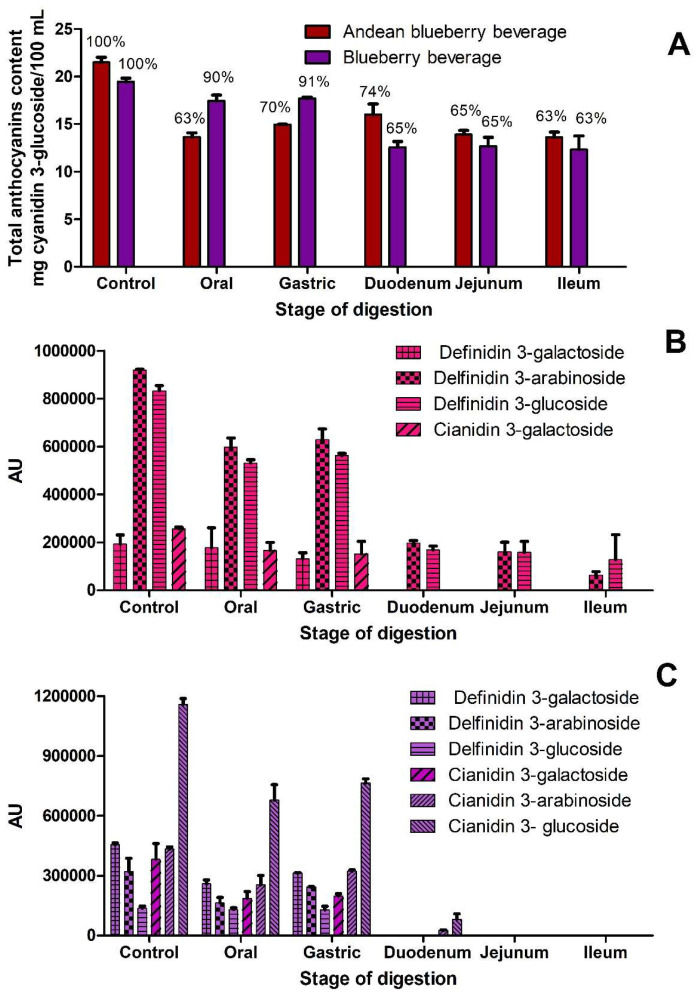
Changes in total anthocyanin content and monomeric anthocyanin profile during simulated in vitro gastrointestinal digestion of optimized fermented whey-based beverages: (**A**) total anthocyanin bioaccessibility, (**B**) monomeric anthocyanin profile of the Andean blueberry (*Vaccinium floribundum* Kunth) beverage, and (**C**) monomeric anthocyanin profile of the blueberry (*Vaccinium corymbosum* L.) beverage. Bars represent mean ± standard deviation.

In contrast, total anthocyanins (Figure 3A) showed lower stability during digestion, particularly under intestinal conditions. The Andean blueberry beverage reached its highest TAC in the duodenal phase, with 16.00 ± 1.10 mg/100 mL (74%), whereas the lowest value was observed in the ileal phase, with 13.61 ± 0.55 mg/100 mL (63%). The blueberry beverage showed its maximum TAC in the gastric phase, with 17.70 ± 0.11 mg/100 mL (91%), followed by a decrease to 12.31 ± 1.45 mg/100 mL (63%) in the final intestinal stage. This behavior agrees with the known pH sensitivity of anthocyanins. Under acidic gastric conditions, anthocyanins are mainly present as flavylium cations, which are more stable and intensely colored. However, when pH increases during intestinal digestion, the flavylium form is converted into quinoidal bases, carbinol pseudobases, chalcones, and degradation products, leading to reduced monomeric anthocyanin recovery [53,54].

The HPLC-DAD profile confirmed that monomeric anthocyanins progressively decreased during digestion (Figure 3B,C), although the degradation pattern depended on the botanical origin and anthocyanin structure. In the Andean blueberry beverage, delphinidin-3-arabinoside and delphinidin-3-glucoside were the predominant compounds in the control, oral, and gastric stages, indicating that the acidic gastric environment partially preserved the delphinidin-rich profile. In the blueberry beverage, cyanidin-3-glucoside was the most abundant anthocyanin before digestion and remained detectable during the oral and gastric stages, together with cyanidin-3-arabinoside and delphinidin derivatives. However, in both beverages, individual anthocyanins declined sharply after transition to the intestinal phase, particularly in the jejunal and ileal stages, where several compounds fell below the quantification limit. This behavior is consistent with the strong pH dependence of anthocyanin chemistry. Under acidic conditions, anthocyanins are mainly stabilized as flavylium cations, whereas at near-neutral intestinal pH, they are rapidly converted into quinoidal bases, carbinol pseudobases, and chalcones, which may subsequently degrade into smaller phenolic acids and aldehydes [53,54]. Similar losses of monomeric anthocyanins during pancreatic–bile digestion have been reported for anthocyanin-rich fruit matrices, confirming that the intestinal phase is the most critical step for anthocyanin stability [55,56]. The presence of pancreatin and bile salts may further accelerate these transformations by modifying solubility, promoting molecular rearrangements, and disrupting weak interactions between anthocyanins and whey proteins. The more pronounced loss of delphinidin derivatives can also be explained by their higher hydroxylation degree in the B ring, which enhances radical-scavenging capacity but increases susceptibility to oxidation compared with less hydroxylated anthocyanins such as cyanidin derivatives [57,58]. Therefore, the decrease in detectable monomeric anthocyanins should not be interpreted as a complete loss of functional activity. Anthocyanin degradation during intestinal digestion may generate smaller phenolic acids, aldehydes, and other low-molecular-weight transformation products that can still retain antioxidant activity. This may partially explain why antioxidant capacity remained high, despite the marked reduction in detectable monomeric anthocyanins during the intestinal stages. Although these degradation products were not individually quantified in the present study, their formation during gastrointestinal digestion of anthocyanin-rich matrices has been widely reported in previous studies [59,60,61]. Thus, the observed decrease likely reflects a combination of pH-induced structural conversion, partial degradation, matrix binding, and formation of non-monomeric or polymeric pigments.

This interpretation is consistent with previous studies showing that anthocyanins can interact with whey proteins through hydrogen bonding, hydrophobic interactions, and electrostatic forces, thereby affecting their stability, color expression, antioxidant capacity, and bioaccessibility [35,36]. In the present study, these interactions provide a reasonable explanatory framework for the observed behavior, although they were not directly characterized.

Overall, the digestion results indicate that fermented whey acted as an active food matrix rather than a passive carrier. The beverages maintained high bioaccessibility of total polyphenols and flavonoids, while antioxidant capacity increased during intestinal digestion, probably due to the combined release of berry-derived phenolics and whey-derived antioxidant peptides. Anthocyanins, in contrast, were more stable under gastric conditions and progressively decreased during intestinal digestion due to pH-dependent structural transformations. These findings highlight the importance of evaluating functional beverages under simulated gastrointestinal conditions, since the biological relevance of phenolic-rich foods depends not only on their initial composition but also on their release, transformation, and stability during digestion.

## 4. Conclusions

This study demonstrated that fermented whey is a suitable and sustainable carrier matrix for developing functional beverages enriched with berry-derived bioactive compounds. The optimized formulations containing 50% berry extract provided the best balance between antioxidant potential, physicochemical stability, and sensory acceptability. Among the evaluated systems, the Andean blueberry (*Vaccinium floribundum* Kunth) beverage showed the best functional performance. This superiority confirms the unique contribution of *V. floribundum* compared with conventional blueberry, particularly because of its higher phenolic and flavonoid contents, stronger ORAC response, and distinctive delphinidin-rich profile. During simulated gastrointestinal digestion, polyphenols and flavonoids remained highly bioaccessible, while ORAC antioxidant capacity increased in the jejunal phase, suggesting a combined contribution of berry phenolics and digestion-derived whey components, with a possible role of antioxidant peptides inferred from the known digestive behavior of whey proteins. In contrast, anthocyanins were more stable under gastric conditions but decreased during intestinal digestion due to pH-dependent structural transformations. Overall, these findings support the valorization of dairy whey as a functional and sustainable food matrix and highlight *V. floribundum* as a promising underutilized berry for the development of antioxidant-rich fermented beverages with improved digestive functionality.

## Figures and Tables

**Table 1:** Physicochemical properties, antioxidant capacity, phenolic compounds, and anthocyanin profile of Andean blueberry (*Vaccinium floribundum* Kunth) and blueberry (*Vaccinium corymbosum* L.) extracts.

Parameter	Andean Blueberry Extract	Blueberry Extract
pH	2.91 ± 0.01 ^a^	3.09 ± 0.01 ^b^
Antioxidant capacity (DPPH) ^1^	2564.91 ± 11.82 ^a^	2497.91 ± 66.88 ^a^
Antioxidant capacity (ORAC) ^1^	5242.89 ± 86.36 ^a^	1953.89 ± 48.18 ^b^
Total polyphenols ^2^	598.92 ± 4.94 ^a^	323.20 ± 7.37 ^b^
Total flavonoids ^3^	406.77 ± 8.59 ^a^	184.16 ± 6.08 ^b^
Total anthocyanins ^4^	32.71 ± 0.77 ^b^	34.68 ± 0.77 ^a^
Delphinidin 3-galactoside ^4^	3.00 ± 0.01 ^a^	5.10 ± 0.01 ^b^
Delphinidin 3-glucoside ^4^	10.64 ± 0.01 ^a^	1.84 ± 0.01 ^b^
Delphinidin 3-arabinoside ^4^	14.47 ± 0.01 ^a^	3.35 ± 0.01 ^b^
Cyanidin 3-galactoside ^4^	4.21 ± 0.01 ^a^	3.32 ± 0.01 ^b^
Cyanidin 3-glucoside ^4^	0.90 ± 0.01 ^b^	13.03 ± 0.01 ^a^
Cyanidin 3-arabinoside ^4^	1.16 ± 0.01 ^b^	5.29 ± 0.01 ^a^

^1^ µmol TE/100 mL; ^2^ mg GAE/100 mL; ^3^ mg QE/100 mL; ^4^ mg C3GE/100 mL. Values are expressed as mean ± standard deviation. Different superscript letters within the same row indicate significant differences between extracts according to Tukey’s test (*p* < 0.05).

**Table 2:** Physicochemical and proximate composition of pasteurized whey, fermented whey, and optimized fermented whey-based beverages formulated with Andean blueberry (*Vaccinium floribundum* Kunth) and blueberry (*Vaccinium corymbosum* L.) extracts.

Parameter	Pasteurized Whey	Fermented Whey	Andean Blueberry Beverage	Blueberry Beverage
pH	6.68 ± 0.01 ^a^	4.52 ± 0.01 ^b^	3.15 ± 0.01 ^d^	3.21 ± 0.00 ^c^
Titratable acidity ^1^	1.47 ± 0.06 ^d^	3.41 ± 0.05 ^a^	1.67 ± 0.02 ^b^	1.54 ± 0.02 ^c^
Moisture ^2^	92.99 ± 0.01 ^b^	93.06 ± 0.01 ^a^	87.56 ± 0.25 ^d^	88.70 ± 0.30 ^c^
Protein ^2^	1.42 ± 0.11 ^a^	1.48 ± 0.27 ^a^	0.44 ± 0.01 ^b^	0.42 ± 0.02 ^b^
Total fat ^2^	1.88 ± 0.15 ^a^	1.52 ± 0.31 ^b^	0.38 ± 0.02 ^c^	0.43 ± 0.02 ^c^
Ash ^2^	0.22 ± 0.01 ^a^	0.28 ± 0.03 ^a^	0.56 ± 0.02 ^b^	0.45 ± 0.02 ^c^
Carbohydrates ^2^	3.49 ± 0.01 ^b^	3.66 ± 0.01 ^a^	11.06 ± 0.06 ^c^	10.00 ± 0.2 ^d^
Lactose ^2^	3.18 ± 0.01 ^a^	1.86 ± 0.05 ^b^	0.97 ± 0.06 ^c^	0.99 ± 0.01 ^c^
Fructose ^2^	ND	ND	4.48 ± 0.14 ^a^	3.93 ± 0.01 ^b^
Glucose ^2^	ND	ND	3.18 ± 0.10 ^b^	3.88 ± 0.02 ª

^1^ g lactic acid/L; ^2^ g/100 mL; ND: not detected. Values are expressed as mean ± standard deviation. Different superscript letters within the same row indicate significant differences among samples according to Tukey’s test (*p* < 0.05).

**Table 3:** Antioxidant capacity, phenolic compounds, and anthocyanin profile of optimized fermented whey-based beverages formulated with Andean blueberry (*Vaccinium floribundum* Kunth) and blueberry (*Vaccinium corymbosum* L.) extracts.

Parameter	Andean Blueberry Beverage	Blueberry Beverage
Antioxidant capacity (DPPH) ^1^	1442.46 ± 12.82 ^a^	335.50 ± 2.34 ^b^
Antioxidant capacity (ORAC) ^1^	2268.97 ± 4.41 ^a^	1508.51 ± 71.26 ^b^
Total polyphenols ^2^	242.60 ± 6.25 ^a^	178.74 ± 5.54 ^b^
Total flavonoids ^3^	137.94 ± 2.76 ^a^	111.29 ± 1.95 ^b^
Total anthocyanins ^4^	21.50 ± 0.51 ^a^	19.46 ± 0.38 ^b^
Delphinidin 3-galactoside	2.15 ± 0.14 ^b^	3.22 ± 0.10 ^a^
Delphinidin 3-glucoside	7.94 ± 0.08 ^a^	1.24 ± 0.06 ^b^
Delphinidin 3-arabinoside	8.96 ± 0.46 ^a^	1.79 ± 0.02 ^b^
Cyanidin 3-galactoside	2.46 ± 0.13 ^a^	2.23 ± 0.01 ^a^
Cyanidin 3-glucoside	ND	7.95 ± 0.18
Cyanidin 3-arabinoside	ND	3.05 ± 0.08

^1^ µmol TE/100 mL; ^2^ mg GAE/100 mL; ^3^ mg QE/100 mL; ^4^ mg C3GE/100 mL; ND: not detected. Values are expressed as mean ± standard deviation. Different superscript letters within the same row indicate significant differences between beverages according to Tukey’s test (*p* < 0.05).

## Data Availability

The original contributions presented in this study are included in the article. Further inquiries can be directed to the corresponding authors.
